# Circadian polymorphisms associated with affective disorders

**DOI:** 10.1186/1740-3391-7-2

**Published:** 2009-01-23

**Authors:** Daniel F Kripke, Caroline M Nievergelt, EJ Joo, Tatyana Shekhtman, John R Kelsoe

**Affiliations:** 1 Department of Psychiatry 0939, University of California, San Diego, La Jolla, CA 92093-0939, USA; 2 Scripps Clinic Sleep Center W207, 10666 North Torrey Pines Road, La Jolla, CA 92037, USA; 3 Department of Neuropsychiatry, Eulji University School of Medicine, Eulji General Hospital, Nowongu Hagedong 280-1, Seoul, Korea

## Abstract

**Background:**

Clinical symptoms of affective disorders, their response to light treatment, and sensitivity to other circadian interventions indicate that the circadian system has a role in mood disorders. Possibly the mechanisms involve circadian seasonal and photoperiodic mechanisms. Since genetic susceptibilities contribute a strong component to affective disorders, we explored whether circadian gene polymorphisms were associated with affective disorders in four complementary studies.

**Methods:**

Four groups of subjects were recruited from several sources: 1) bipolar proband-parent trios or sib-pair-parent nuclear families, 2) unrelated bipolar participants who had completed the BALM morningness-eveningness questionnaire, 3) sib pairs from the GenRed Project having at least one sib with early-onset recurrent unipolar depression, and 4) a sleep clinic patient group who frequently suffered from depression. Working mainly with the SNPlex assay system, from 2 to 198 polymorphisms in genes related to circadian function were genotyped in the participant groups. Associations with affective disorders were examined with TDT statistics for within-family comparisons. Quantitative trait associations were examined within the unrelated samples.

**Results:**

In *NR1D1*, rs2314339 was associated with bipolar disorder (P = 0.0005). Among the unrelated bipolar participants, 3 SNPs in *PER3 *and *CSNK1E *were associated with the BALM score. A *PPARGC1B *coding SNP, rs7732671, was associated with affective disorder with nominal significance in bipolar family groups and independently in unipolar sib pairs. In *TEF*, rs738499 was associated with unipolar depression; in a replication study, rs738499 was also associated with the QIDS-SR depression scale in the sleep clinic patient sample.

**Conclusion:**

Along with anti-manic effects of lithium and the antidepressant effects of bright light, these findings suggest that perturbations of the circadian gene network at several levels may influence mood disorders, perhaps ultimately through regulation of MAOA and its modulation of dopamine transmission. Twenty-three associations of circadian polymorphisms with affective symptoms met nominal significance criteria (P < 0.05), whereas 15 would be expected by chance, indicating that many represented false discoveries (Type II errors). Some evidence of replication has been gathered, but more studies are needed to ascertain if circadian gene polymorphisms contribute to susceptibility to affective disorders.

## Background

The idea that circadian rhythms had some role in affective disorders arose from clinical observations of their altered sleep-wake cycles, the cyclicity of the symptoms, and early work by pioneering researchers [1-4]. Attempts to identify circadian abnormalities in depressed and bipolar patients by physiologic means have yielded somewhat inconsistent and disappointing results [5-7]. However, the now-proven efficacy of bright light treatment as well as a broader range of effective interventions in the circadian system provide strong evidence that circadian rhythms are somehow involved in the pathophysiology of affective disorders [8].

The effects of light treatment, along with the symptom development of seasonal affective disorder (often a bipolar phenotype), might suggest that mechanisms which trigger mood swings in humans resemble the circadian-controlled photoperiodic mechanisms governing mammalian seasonality [9]. Recently, a number of studies of nocturnal and diurnal rodents have demonstrated influences of photoperiod upon animal models of depression [10-12]. Several reports have presented rationales and preliminary suggestive evidence that circadian system genetic abnormalities might contribute to affective disorders [13-18]. In addition, accumulating evidence indicates that heritable circadian disorders such as delayed sleep phase disorder are comorbid with depression [19]. This may suggest that there are genetic polymorphisms in the circadian system which confer susceptibility both to depression and to delayed sleep phase disorder or its converse, advanced sleep phase disorder.

Genome-wide association studies of bipolar disorder have given no substantial support for a role of the circadian system [20,21], although in one study, *VGCNL1*, a gene which may have a circadian role, came close to genome-wide significance [22]. Genome-wide studies, however, are designed to detect common allelic variants of small effect, and do not exclude other types of gene effects, such as rare variants of strong effect.

Though the genome-wide association method may eventually replace the testing of candidate genes, we have thought it worthwhile to survey likely single nucleotide polymorphisms (SNPs) in the set of genes which form the circadian system through complex interactions. Most of the SNPs we have considered have not been tested directly in whole-genome association studies. Moreover, in some models, we have used transmission disequilibrium tests (TDT) with parent-proband trios or affected sib pairs which eliminate population stratification as a potential source of false-negative results. Here we report results of 4 ongoing studies which provide some cross-replication, and taken together, suggest that several circadian polymorphisms are associated with phenotypes related to affective disorders.

## Methods

We describe 4 complementary studies, assembled to provide replication and to clarify what aspects of circadian polymorphisms may be relevant to both bipolar and unipolar affective disorders.

### Bipolar probands and families

From probands with bipolar disorder, DNA samples from 444 nuclear families were assembled including 561 affected offspring. These were largely proband-parent trios or affected sib pairs with parents. These nuclear families were obtained primarily from two different samples. The first was a set of families collected as part of a three site consortium (UCSD, U. Cincinnati, and U. British Columbia) for linkage studies in extended pedigrees. The remainder of the families came from waves 1–4 of the NIMH Genetics Initiative for Bipolar Disorder Collection. Both family sets and the ascertainment and diagnostic methods employed have been described in detail elsewhere [23,24]. For this analysis, we included bipolar type 1 disorder, bipolar type 2 disorder, and schizoaffective (bipolar type) patients as affected participants. Although the TDT is not subject to an increased type one error rate due to population stratification, only self-identified Caucasians were included in this analysis.

Single-nucleotide polymorphisms were assayed with 6 reagent pools targeting 45–48 SNPs, using the SNPlex™ Genotyping System with an ABI 3730 48-capillary DNA analyzer according to the manufacturer's directions (Applied Biosystems, Foster City, California). Technically satisfactory genotypes with sufficient heterozygosity for analysis were obtained for 197 SNPs [see Additional file 1]. In addition, a polymorphic repeat region with four or five copies of a 54 bp repetitive sequence in exon 18 of the *PER3 *gene was examined [25,26]. PCR of the polymorphic area was performed using the primers: 6-FAM AGGCAACAATGGCAGTGAG fluorescently labeled, and Rev AATGTCTGGCATTGGAGTTTG. Products of 309 bp and 363 bp were distinguished by gel electrophoresis, using the 6-Fam fluorescent label on the forward primer to determine fragment size. PLINK v1.03 [27] was used to test for HWE, and transmission disequilibrium from parent to affected child was tested using a transmission-disequilibrium test (TDT). Empirical p-values were generated using the max(T) permutation approach for pointwise estimates (EMP1) as well as corrected for all comparisons (EMP2). Compared to a conservative Bonferroni correction for multiple comparisons, a global permutation test is a more powerful approach for candidate gene studies as it considers the correlation structure between SNPs in LD with each other. Correlation between SNPs (LD structure) was assessed with HaploView 4.1. (Broad Institute, Cambridge, MA).

### Bipolar probands and the morningness-eveningness quantitative trait

A group of 130 unrelated research volunteers completed the Basic Language Morningness (BALM) Scale, a 13-item multiple-choice questionnaire designed to distinguish participants with high, normal, or low "morningness" [28]. These subjects were recruited at UCSD for genetic studies of bipolar disorder, but they were not primarily parts of family groups and only 5 were also included in the TDT family sample. Of the 130 subjects with available BALM data, according to research diagnoses, 82% were Bipolar Type I (not necessarily manic at the time), 3 had had unipolar major depression, one was schizoaffective, and the rest had no psychiatric diagnosis. Those with high BALM scores tend to go to bed early and arise early: in the extreme, they may suffer from advanced sleep phase disorder. Those with low BALM scores tend to go to bed late and to arise late in the morning: with extremely low scores, they may suffer from delayed sleep phase disorder. This quantitative trait, thought to reflect control by circadian "clock" genes, is roughly 50% heritable [29-31]. The 6th (most recent) SNPlex™ pool was assayed for each participant, but the other assay reagent pools were not available. Of the 48 SNPs in the pool, 44 were successfully assayed and 30 passed quality control. With PLINK [27], quantitative trait associations (additive model) were performed and empirical p-values estimated based on the Wald-statistic (t-distribution). To correct for population stratification, subjects were grouped into self-identified Caucasians (n = 95) and others (n = 35) and permutations were performed within these two groups using the max(T) permutation approach for pointwise estimates (EMP1) as well as corrected for multiple comparisons (EMP2).

### Unipolar major depression affected sibling pairs

Families with probands with recurrent early-onset unipolar depression were recruited by the GenRED project [32]. These subjects were ascertained as part of a multi-site consortium to conduct linkage studies of major depression, and diagnoses made using a standardized best estimate method as previously described. Through the National Institute of Mental Health Human Genetics Initiative, DNA from 150 GenRED sibling pairs with at least one affected sibling was kindly supplied by the Rutgers University Cell & DNA Repository. These samples were also assayed with SNPlex pools 5 and 6 (88 SNPs), resulting in high-quality genotypes of 63 SNPs. Using PLINK, family-based sib-TDT (DFAM) analyses were computed including 298 individuals in 149 families (89 concordant and 60 discordant sib-pairs). As in the other analyses, empirical p-values were generated using the max(T) permutation approach for pointwise estimates (EMP1) as well as corrected for multiple comparisons (EMP2). To safeguard against spurious associations due to population stratification, a TDT was used in this sample approximately 95% of European origin [32].

### Sleep Clinic sample

Patients of the Scripps Clinic Sleep Center who underwent polysomnography or some other form of sleep recording were invited to participate in a descriptive genetic study. They consented to contribute saliva DNA samples and a research questionnaire, which included the BALM morningness-eveningness scale and the QIDS-SR self-rated depression scale [33]. This sample was 90% Caucasian by self-report. DNA was extracted and genotyped for 360 participants in the DNA Core Laboratory of the Molecular and Experimental Medicine division of the Scripps Research Institute. The alleles of rs2314339 and rs738499 were identified by allele-specific oligonucleotide hybridization [34].

### Ethical guidelines

Since the DNA samples were collected from many sources, the original publications should be consulted for information concerning institutional review boards. In general, the data were collected in accord with the principles of the Declaration of Helsinki.

## Results

### Transmission disequilibrium in families of bipolar probands

Of approximately 260 SNPs assayed in the SNPlex pools or by gel electrophoresis, 212 polymorphisms were successfully genotyped. Of these, 198 polymorphisms yielded polymorphic genotypes of acceptable quality [see Additional file 1]. The TDT was applied to these polymorphisms, located in or near 26 genes associated with the circadian system. As shown in Table 1, 17 polymorphisms had nominal P values < 0.05, modestly exceeding the random expectation of 10 of 198 polymorphisms.

**Table 1:** Polymorphisms associated with bipolar disorder

**Gene**	**CHR**	**SNP**	**A1**	**A2**	**MAF**	**T**	**U**	**OR**	**CHISQ**	**P**	**EMP1**	**EMP2**
***NPAS2***	2	rs1562313	A	G	0.213	208	165	1.261	4.957	0.0260	0.0484	0.9803
***PER2***	2	rs4663868	T	C	0.071	85	54	1.574	6.914	0.0086	0.0171	0.7330
***PER2***	2	rs2304669	G	A	0.164	131	167	0.784	4.349	0.0370	0.0416	0.9945
***PER2***	2	rs2304672	G	C	0.073	88	55	1.600	7.615	0.0058	0.0123	0.5972
***CLOCK***	4	rs3805148	C	A	0.366	255	197	1.294	7.442	0.0064	0.0092	0.6291
***CLOCK***	4	rs3736544	A	G	0.373	229	280	0.818	5.110	0.0238	0.0241	0.9728
***CLOCK***	4	rs12504300	C	G	0.282	188	138	1.362	7.669	0.0056	0.0094	0.5775
***CLOCK***	4	rs4864542	G	C	0.364	273	212	1.288	7.672	0.0056	0.0102	0.5759
***CLOCK***	4	rs12648271	C	G	0.285	227	182	1.247	4.951	0.0261	0.0369	0.9806
***CLOCK***	4	rs6850524	C	G	0.424	232	282	0.823	4.864	0.0274	0.0322	0.9840
***PPARGC1B***	5	rs7732671	C	G	0.068	65	42	1.548	4.944	0.0262	0.0213	0.9811
***PER1***	17	rs2585405	G	C	0.104	86	117	0.735	4.734	0.0296	0.0457	0.9881
***THRA***	17	rs939348	T	C	0.279	188	239	0.787	6.091	0.0136	0.0224	0.8729
***NR1D1***	17	**rs2314339**	T	C	0.130	90	147	0.612	13.710	0.0002	0.0005	**0.0338**
***NR1D1***	17	rs2071427	A	G	0.256	191	256	0.746	9.452	0.0021	0.0019	0.2852
***NR1D1***	17	rs2269457	G	A	0.239	171	214	0.799	4.803	0.0284	0.0292	0.9861
***CSNK1D***	17	rs4510078	A	G	0.021	18	38	0.474	7.143	0.0075	0.0175	0.6942

GENE: NCBI gene symbol. CHR: chromosome number. SNP: dbSNP symbol. A1 & A2: minor and major allele nucleotides. MAF: minor allele frequency. T: number of transmissions of the rare allele. U: number of untransmitted rare alleles. OR: odds ratio of TDT. CHISQ: Chi Square from TDT. P: probability of Chi Square. EMP1: empirical probability from simulation by PLINK. EMP2: corrected empirical P (Max T/familywise).

The strongest association with bipolar disease was found with *NR1D1 *(Rev-erb-alpha, OMIM 602408). Using a permutation procedure to correct for multiple comparisons, rs2314339, an intronic SNP, showed a significant association (odds ratio 0.61, P(_nominal_) < 0.0005, P(_corrected_) < 0.035). Using a false discovery rate threshold of 5%, rs2314339 was significantly associated with disease status (q-value < 0.05) [35]. In addition, two SNPs within this gene and one SNP within the nearby *THRA *were nominally significant (p < 0.05, Table 1). The SNPs most strongly associated in this region were moderately correlated with one another (pairwise r-squared between rs2314339 and rs2071427 = 0.26; rs2314339 and rs2269457 = 0.29; rs2314339 and rs939348 = 0.27).

Suggestive evidence for association with bipolar disease was also found for the *CLOCK *gene (OMIM 601851). Thirteen SNPs were investigated in this gene, and six of them were nominally significant by the EMP1 criterion, the most significant being rs3805148 (p = 0.0092) and rs12504300 (p = 0.0094) (Table 1). These 6 SNPs are all in linkage disequilibrium (pairwise r-squared 0.23–0.99) in a single 75 KB linkage block which covers almost all the gene. They form a common, overtransmitted haplotype with a frequency of 28.2 percent (234.1:181.5 T:U, P(_nominal_) < 0.01). The often-discussed T3111C SNP in the 3' UTR (rs1801260) [36,37] was not in close linkage disequilibrium with these 6 SNPs (largest pairwise r-squared < 0.22), and it was not significantly associated with bipolar disease (P > 0.69).

In addition, modest evidence for association to bipolar disease was also found for *PER2 *(OMIM 603426) with 3 of the 15 tested *PER2 *SNPs nominally significant (rs4663868: p < 018, rs2304672: p < 0.013; pairwise r-squared = 0.93; rs2304669: p < 0.042, not in LD with the other 2 SNPs).

### SNPs associated with the BALM in bipolars

Of the SNPs in bipolars for whom BALM data were available, 30 yielded acceptable genotypes which were sufficiently polymorphic for analysis [see Additional file 2]. Of these, three relatively rare SNPs were associated with BALM values with significance at a P < 0.05 criterion after correction for multiple comparison (Table 2). Results for additive and dominant models were similar, since there were few homozygotes of the rare allele for these rare SNPs (data not shown).

**Table 2:** SNPs associated with the BALM among bipolar participants

**Gene**	**CHR**	**SNP**	**A1**	**A2**	**MAF**	**BETA**	**R2**	**EMP1**	**EMP2**	**genotype freq**
*PER3*	1	rs228697	G	C	0.083	-6.36	0.0913	0.0008	0.0150	4/15/110
*CSNK1E*	22	CSNK1E28266	A	G	0.025	13.67	0.0785	0.0008	0.0467	0/5/119
*CSNK1E*	22	CSNK1E27740	T	G	0.028	12.45	0.0753	0.0010	0.0506	0/6/121

CHR: chromosome. A1 & A2: minor and major allele nucleotides. MAF: minor allele frequency. R^2^: the correlation squared. EMP1: pointwise empirical P value. EMP2: corrected empirical P value from max/(T)/familywise.

The *PER3 *nonsynonymous coding SNP Ala856Pro (rs228697) was associated with the BALM with R^2 ^= 0.091, P = 0.0008 in an additive model. The presence of the rare SNP allele was associated with greater eveningness (i.e., a lower BALM morningness score): a mean BALM of 26 for 4 homozygotes for the minor allele, a mean BALM of 31 for 15 heterozygotes, and a mean BALM of 38 for the 110 homozygotes with the common allele. Although the genotypes barely failed Hardy-Weinberg equilibrium (P_nominal _< 0.04) in this population, this SNP showed no deviation from HWE in the simultaneously assayed bipolar TDT sample, indicating good genotyping quality.

Two intronic SNPs in *CSNK1E *were associated with the BALM with nominal P < 0.001 in an additive model, associated with almost 8% of the variance. The two SNPs are 526 nucleotides apart and essentially in perfect linkage disequilibrium. For one subject, one of the linked SNPs could not be genotyped. The mean BALM scores were 49–50 for heterozygotes (high morningness) and 36 for homozygotes with the common allele. Four or 5 of the heterozygotes had a BALM ≥ 50, the 95^th ^percentile, indicating extreme morningness, but one had the minimum possible BALM, indicating extreme eveningness. Thus, the few heterozygotes were heterogeneous in BALM morningness-eveningness. These polymorphisms have been submitted to NCBI as nucleotides 27740 and 28266 in Core Nucleotide Report EF015901 (available at ).

### Unipolar major depression sib pairs

There were 89 sib pairs concordant for unipolar major depressive disorder and 60 discordant sib pairs with one twin having no mental illness. They were genotyped for 61 SNPs which proved sufficiently heterozygous and one repeat region in *PER3*. Of these, 2 reached nominal significance (Table 3). In the promoter region of TEF, rs738499 was associated with MDD by sib-TDT with P = 0.012. The minor G allele was protective. Also, rs7732671 in *PPARGC1B *was associated with P = 0.023, with the minor allele being associated with depression. Neither SNP was significant after correction for multiple comparison (EMP2) [see Additional file 3].

**Table 3:** SNPs associated with unipolar recurrent major depression

**Gene**	**CHR**	**SNP**	**A1**	**A2**	**MAF**	**OBS**	**EXP**	**EMP1**	**EMP2**
*PPARGC1B*	5	rs7732671	C	G	0.092	14	10	0.0232	0.3148
*TEF*	22	rs738499	G	T	0.334	35	41.5	0.0114	0.2123

CHR: chromosome. A1 & A2: minor and major allele nucleotides. MAF: minor allele frequency. OBS: number of observed minor alleles. Exp: number of expected minor alleles. EMP1: pointwise empirical P value. EMP2: corrected empirical P value from max/(T)/familywise.

### Sleep clinic patients

Many Sleep Clinic patients report some degree of depression. Their QIDS-SR averaged 6.9 (in the mildly depressed range) with SD 3.9. Also, 16.2% scored ≥ 10, in the moderately depressed range. In an attempt to replicate results from other subject groups, 2 SNPs were examined: rs738499 and rs2314339. The number of the less-common G alleles in the *TEF *T>G SNP rs738499 was correlated with the QIDS-SR, R_s _= -0.165 (P = 0.001, Spearman Rank Order Correlation). The negative correlation suggests that the G allele was associated with normal mood and might account for about 3% of the variance. Neither rs738499 nor rs2314339 were correlated with the BALM nor was rs2314339 correlated with the QIDS-SR.

## Discussion

In four different analyses, circadian gene polymorphisms were studied for association with three phenotypes: bipolar disorder, unipolar depression (major depressive diagnosis or rating-scale quantitative trait), and morningness-eveningness (which shares comorbidity with major depression) [19]. In 294 tests of association, 23 different associations met the nominal significance criterion of P < 0.05, whereas 15 such associations would have been anticipated by random chance. It is plausible that most of the nominal associations were due to random chance (false discovery), but at least 5 appeared associated with these affective phenotypes with sufficient evidence of reliability to be considered suggestive.

The intronic SNP rs2314339 in *NR1D1 *met false discovery and empirical family-wise criteria for significant association with the transmission of bipolar disorder (P = 0.0005). The TDT analysis should be free from biases due to racial stratification. The same SNP was associated with delayed sleep phase disorder cases in an unpublished case-control sample, but significance was not sustained in the case-control sample after preliminary control for racial stratification. In both analyses, the more common allele was associated with the disorder and the less common allele was associated with control or normal health. The DSPD data offered at best only an indirect kind of replication because of the difference in phenotype between bipolar disorder and DSPD, especially considering that in our DSPD sample, we had found comorbidity with unipolar depression but not with bipolar disorder [19]. Moreover, rs2314339 has been tested in some whole genome association studies of bipolar disorder, but we are unaware that any suggestive association has been found in such studies. Another nuclear receptor, *NR2E1*, has been reported to be associated with bipolar disorder in a case-control study [38]: there has been brief mention that *NR2E1 *and *NR1D1 *may interact in the development of photoreceptors.

**Figure 1:**
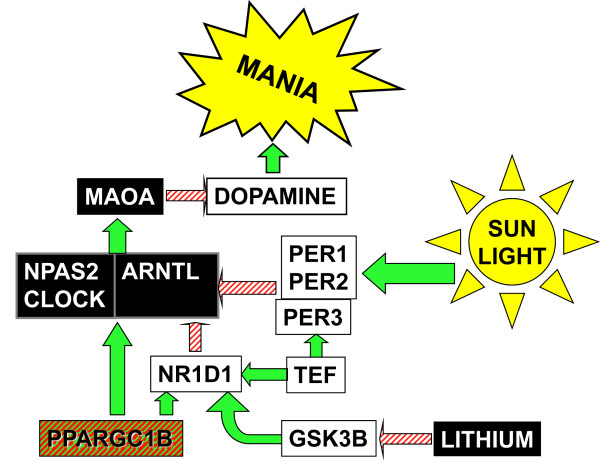
**Model relating sunlight, lithium, and circadian genes to MAOA and mania**. This model relates sunlight and lithium to components of the circadian gene system, to MAOA (monoamine oxidase A), dopamine, and resultant stimulation of mania. Green solid arrows represent interactions which promote the function of the affected component. Red striped arrows represent inhibition of the function of the affected component. Components in white boxes hypothetically promote mania. Components in black boxes hypothetically inhibit mania. The red-green striped box for PPARGC1B suggests its opposing roles in possibly stimulating both ARNTL and NR1D1, whereas NR1D1 then inhibits ARNTL. The positive feedback of ARNTL-CLOCK and ARNTL-NPAS2 heterodimers on NR1D1, TEF, PER1, PER2, and PER3 was omitted from the diagram for simplicity, along with many other components and interactions within the circadian system.

NR1D1 (OMIM 602408) is a key element of a unique circadian feedback loop, in which it inhibits transcription of *ARNTL *(BMAL1) by inhibitory binding at *ARNTL *RORE promoter sites [39]. *NR1D1 *may similarly inhibit transcription of *CLOCK *and *NPAS2*, the proteins of which activate *ARNTL *by binding as heterodimers. *NR1D2 *possibly has a similar role (OMIM 602304). These mechanisms may be particularly relevant to bipolar disorder, since it has been suggested that a mutation of *CLOCK *(which produces hyperactivity) may be an animal model for bipolar mania [40]. A knockout of *ARNTL*, on the other hand, reduces activity, though that can be largely restored by replacing ARNTL function in muscle [41]. ARNTL heterodimers with NPAS2 may bind to promoter eboxes of *MAOA*, thus promoting inactivation of dopamine, and thus inhibiting the pro-manic effects of dopamine [42-44]. Oddly enough, the ARNTL-CLOCK heterodimer was not demonstrated to have a similar effect on *MAOA*, though ARNTL-CLOCK would be expected to act on the same e-boxes in the *MAOA *promoter. Lithium, a primary medication for treatment of bipolar disorder, promotes degradation of NR1D1 through inhibition of GSK3, whereas GSK3 phosphorylation stabilizes NR1D1 [45]. These interactions are modeled in Fig. 1.

*NR1D1 *or Rev-erb-alpha is so-called, because it is transcribed in the reverse direction and overlaps the 3' end of *THRA*, an important thyroid nuclear receptor. It is interesting that thyroid dysfunction becomes most prominent among bipolar patients after treatment with lithium [46]. Also, thyroid (T3) augmentation is useful for treating depression [47]. Thyroid has been used for periodic catatonia (perhaps a form of bipolar disorder) since the 1930's [1]. Considering that the intronic location of rs2314339 indicates no obvious functional role, we suspect that this SNP might be in linkage disequilibrium with some nearby polymorphism with a key functional effect. As linkage disequilibrium for rs2314339 extends through most of *NR1D1 *and to the 3' end of *THRA*, the functional element could plausibly be situated in either gene.

A coding SNP in *PPARGC1B*, Pro203Ala, rs7732671, was over-transmitted to bipolar probands with P < 0.05 and odds ratio 1.55. By itself, we might regard this isolated finding as statistically unimpressive and plausibly a false positive. However, the same SNP was associated with unipolar depression with an odds ratio of 2.12 (P < 0.025). The nominally significant association in both completely separate subject sets with odds ratios in the same direction provides suggestive evidence for a reliable association, especially since neither statistical result is sensitive to false positive results from population stratification. The common allele of this SNP has been associated with obesity (OMIM 608886). A paralogue gene, *PPARGC1A*, is a regulator of *ARNTL *and additionally functions through regulation of NR1D1 and NR1D2 effects on *ARNTL *[48]. PPARGC1A possibly binds to RORE sites both on *NR1D1 *and on *ARNTL*, and PPARGC1B may act similarly, perhaps providing a partial explanation for effects on both mania and depression, seeming opposites which are both aspects of bipolar disorder (Fig. 1). Thus, there may be a convergence of pathways. A number of SNPs in *PPARGC1B *achieved nominal significance in a case-control study which included some of these same bipolar subjects, but none approached Bonferroni criteria [22].

The TDT association of 6 *CLOCK *SNPs with bipolar disorder was intriguing, and appeared consistent with the claim that a *CLOCK *mutation in mice produces a mouse analog of mania [40]. Although nominally significant, associations with these SNPs could all represent false-positive statistical findings. One of the 6 SNPs, rs6850524 was also found to be suggestively associated with bipolar disorder in analyses using a partially-overlapping subject sample [17]. Also, *CLOCK *SNP rs2412648 (P < 0.05 by Chi Square, P > 0.05 by EMP1 in our sample) was part of a suggestively-associated haplotype [17]. It would be conceivable that many SNPs in the *CLOCK *gene, each with a small effect impairing the gene, could in combination make a substantial contribution of bipolar susceptibility. Because the *CLOCK *gene, extending over roughly 114,338 base pairs, displays high linkage disequilibrium throughout its considerable length, it is possible that the most functional polymorphism has not yet been recognized. Similarly, though various associations with several bipolar phenotypes such as recurrence rates and sleep disturbances have been reported with rs1801260, the T3111C SNP in the 3'UTR region of *CLOCK *[36,37], it is possible that rs1801260 is not the most functional polymorphism in linkage disequilibrium. In our TDT analysis and in BALM studies, rs1801260 was not associated with bipolar disorder, nor was it associated with the morningness-eveningness dimension, as had been reported in other data [36].

In 1978, at a time when the gene causing the *Drosophila PER *mutant had not yet been identified and the presence of 3 human homologues was unknown, the first author hypothesized that bipolar disorder might be caused by mutation of a homologue of the *PER *gene [49]. The three SNPs in *PER2 *and rs2585405 in *PER1*, which were over-transmitted or under-transmitted to bipolar probands with nominal significance, gave weak support to this archaic hypothesis, but certainly suggested no major role for the *PER *genes in bipolar disorder. On the other hand, associations of affective symptoms with other polymorphisms provided some of the strongest evidence that the circadian system has a role in affective disorders. The lack of consistency in results for the *PER *homologues in different groups was somewhat disappointing and reminds us that these associations may be false positive results. It would appear that additional large and independent samples must be studied to determine if the *PER *genes have a real role in human affective disorders. Bright light, which promotes mania, tends to promote transcription of the *PER1 *and *PER2 *genes [50], which may then inhibit the action of the ARNTL-NPAS2 heterodimer in stimulating *MAOA *(Fig. 1). Thus, this pathway may also be consistent with our model in Fig. 1. However, the model ignores numerous problems and complexities of circadian regulation and fails to incorporate the dynamic circadian fluctuations or the photoperiodic interactions among circadian system components. We also have not explained how the observed differences in genetic background might produce the distinctive phenotypes among patients with unipolar and bipolar disorders, nor have we suggested the mechanism by which the same genetic background creates susceptibility in bipolar patients to both mania and depression.

The *TEF *promoter SNP rs738499 had a statistically unimpressive (P_nominal _= 0.023) association with unipolar recurrent depression, with the less common G allele being associated with healthy mood. A similar association with the QIDS-SR in the Sleep Center sample provided a degree of replication (P < 0.001), using independent subjects, independent methods, and a different lab and assay procedure. We had selected rs738499 for genotyping based on a report that rs5996091, a SNP over 500,000 nucleotides remote from *TEF*, was highly correlated with *TEF *expression with R^2 ^= 0.43 [51], and we had noted that of HapMap SNPs nearer *TEF*, rs738499 had the highest linkage with rs5996091 as well as a likely location in the promoter. By stimulatory binding at D-box promoter sites, TEF may promote transcription of *NR1D1*, *NR1D2*, and the *PER *genes, actions which might be supposed to stimulate mania or counter depression (Fig. 1). However, we do not know if the rs738499 minor allele promotes or inhibits *TEF *transcription.

Two intronic SNPs in *CSNK1E *were associated with the BALM among bipolar subjects, meeting Bonferroni criteria for significance. The two SNPs were in virtually perfect linkage disequilibrium with each other. However, among 26 SNPs in *CSNK1E *identified by resequencing 12,173 nucleotides of exonic, intronic, and promoter regions of the gene, no other SNPs were found to be in substantial linkage disequilibrium with these two SNPs (see Core Nucleotide Report EF015901). Surprisingly, although 4 of the 6 participants from the bipolar sample with these two SNPs displayed extreme morningness, one subject of the 6 had a score at the opposite end of the morningness-eveningness scale. Both SNPs were under-transmitted to bipolars (NS). CSNK1E phosphorylates several of the circadian proteins including the PER proteins and ARNTL, and may even have differential effects on phase adjustment depending on which phosphorylation sites are intact on various substrates [52,53]. CSNK1D has a somewhat similar role. Note that one *CSNK1D *SNP was nominally associated with bipolar disorder, and the *CSNK1D *region on 17q achieved a maximum LOD score of 3.63 in a bipolar association study [54]. However, since the two *CSNK1E *SNPs and the *PER3 *SNP associated with the BALM scale of morningness-eveningness were not associated with the bipolar and unipolar psychiatric phenotypes, it seems that the circadian polymorphisms which are related to affective disorders do not influence affective state simply through effects on circadian phase, e.g., Fig. 1 does not suggest that the effects are mediated through circadian phase change.

It is important to review some suggestive findings reported elsewhere which were not replicated in these analyses. We were not able to confirm the suggestive evidence we had earlier reported that haplotypes in *ARNTL *and *PER3 *were associated with bipolar disorder [15]. Those haplotype associations had previously fallen short of Bonferroni criteria. We have also examined certain candidates for association proposed by Mansour and colleagues [13,16]. These included rs7107287, rs4757142, and rs1982350 in *ARNTL*, rs2859387 in *PER3*, and rs2291738 and rs2279665 in *TIMELESS*. None of these reached nominal significance of P < 0.05 in our TDT analyses. The rs11541353 SNP in *NPAS2 *and rs2290035 in *ARNTL*, reported to be associated with seasonal affective disorder [55,56], were not significantly associated with bipolar disorder in our families by TDT. In *PER3*, rs10462020 was not associated with bipolar disorder or unipolar depression. Also, we have not yet demonstrated any association of the *PER3 *repeat described by Archer et al. with affective disorders [26].

## Conclusion

In summary, we found several suggestive associations of circadian gene polymorphisms with affective disorders, some of which we were able to partially replicate. The association of *NR1D1 *rs2314339 with bipolar disorder and DSPS and the association of *PPARGC1B *Pro203Ala, rs7732671, with both bipolar and unipolar affective disorders appear the most likely to prove reliable. The association of *TEF *rs738499 with unipolar depression may also prove reliable. Two intronic SNPs in *CSNK1E *were associated with the BALM in bipolars, but the inconsistent directions of association stimulate some reserve, and these SNPs were not directly associated with affective diagnoses or symptoms. Each of these leads should be pursued. When genotyping of ancestry-informative markers becomes available, our correlations of the BALM with genotypes should be controlled for population stratification, which is a potential problem in the Sleep Center sample as well. Replication and extension of these results in larger independent samples is needed before the importance of circadian polymorphisms in affective syndromes can be verified. If the findings are confirmed, they will suggest that bipolar and unipolar affective disorders have at least one common genetic susceptibility factor, but several which are distinct.

## Competing interests

JRK is a founder and holds equity in Psynomics, Inc. The terms of this arrangement have been reviewed and approved by UCSD in accordance with its conflict of interest policies. The other authors declare that they have no competing interests.

## Authors' contributions

DFK suggested the primary hypotheses, supervised recruitment of the Sleep Clinic sample, selected the polymorphisms to be assayed, and wrote much of the manuscript. CMN contributed to the hypotheses and design of the study, performed most of the statistical analyses, and wrote parts of the manuscript. EJJ assembled the data for the non-related bipolar sample and critiqued the manuscript. TS helped manage and store the DNA samples, performed the SNPlex assays, assembled assay results, and wrote portions of the manuscript. JRK developed the team collecting and assembling the UCSD bipolar samples, participated in the NIMH Bipolar Disorder Genetics Initiative, developed the assay laboratory, contributed to design, and wrote parts of the manuscript. All authors read and approved the final manuscript.

## Supplementary Material

Additional file 1 **SNPs associated with transmission of bipolar disorder**. 198 polymorphisms entered into TDT tests for association with bipolar disorder are listed. SNPs nominally significant are highlighted in yellow and the SNP significant after control for multiple comparisons is highlighted in green. Click here for file

Additional file 2 **SNPs of bipolar participants associated with the BALM**. 30 SNPs tested for association with the BALM morningness-eveningness scale are listed, with the SNPs significant after control for multiple comparisons highlighted in green. Click here for file

Additional file 3 **SNPs associated with unipolar depression**. 62 polymorphisms tested for association with recurrent unipolar depression are listed, with SNPs nominally significant highlighted in yellow. Click here for file

## References

[B1] Gjessing RR (1976). Contribution to the Somatology of Periodic Catatonia.

[B2] Jenner FA, Gjessing LR, Cox JR, vies-Jones A, Hullin RP, Hanna SM (1967). A manic depressive psychotic with a persistent forty-eight hour cycle. Br J Psychiatry.

[B3] Halberg F (1967). Physiologic considerations underlying rhythmometry, with special reference to emotional illness. Symposium on Biological Cycles and Psychiatry. Symposium Bell-Air III.

[B4] Richter CP (1965). Biological Clocks in Medicine and Psychiatry.

[B5] Nurnberger JI, Adkins S, Lahiri DK, Mayeda A, Hu K, Lewy A, Miller A, Bowman ES, Miller MJ, Rau NL (2000). Melatonin suppression by light in euthymic bipolar and unipolar patients. Arch Gen Psychiatry.

[B6] Salvatore P, Ghidini S, Zita G, De PC, Lambertino S, Maggini C, Baldessarini RJ (2008). Circadian activity rhythm abnormalities in ill and recovered bipolar I disorder patients. Bipolar Disord.

[B7] Ahn YM, Chang J, Joo YH, Kim SC, Lee KY, Kim YS (2008). Chronotype distribution in bipolar I disorder and schizophrenia in a Korean sample. Bipolar Disord.

[B8] Wirz-Justice A (2007). Chronobiology and psychiatry. Sleep Med Rev.

[B9] Kripke DF, Wurtman RJ, Baum MJ, Potts JT Jr (1985). Therapeutic effects of bright light in depressed patients. The Medical and Biological Effects of Light.

[B10] Prendergast BJ, Nelson RJ (2005). Affective responses to changes in day length in Siberian hamsters (Phodopus sungorus). Psychoneuroendocrinology.

[B11] Einat H, Kronfeld-Schor N, Eilam D (2006). Sand rats see the light: short photoperiod induces a depression-like response in a diurnal rodent. Behav Brain Res.

[B12] Yilmaz A, Aksoy A, Canbeyli R (2004). A single day of constant light (L/L) provides immunity to behavioral despair in female rats maintained on an L/D cycle. Prog Neuropsychopharmacol Biol Psychiatry.

[B13] Mansour HA, Monk TH, Nimgaonkar VL (2005). Circadian genes and bipolar disorder. Ann Med.

[B14] Nievergelt CM, Kripke DF, Remick RA, Sadovnick AD, McElroy SL, Keck PE, Kelsoe JR (2005). Examination of the clock gene *Cryptochrome 1 *in bipolar disorder: mutational analysis and absence of evidence for linkage or association. Psychiatr Genet.

[B15] Nievergelt CM, Kripke DF, Barrett TB, Burg E, Remick RA, Sadovnick AD, McElroy SL, Keck PE, Schork NJ, Kelsoe JR (2006). Suggestive evidence for association of the circadian genes *Period3 *and *Arntl *with bipolar disorder. Am J Med Genet B Neuropsychiatr Genet.

[B16] Mansour HA, Wood J, Logue T, Chowdari KV, Dayal M, Kupfer DJ, Monk TH, Devlin B, Nimgaonkar VL (2006). Association study of eight circadian genes with bipolar I disorder, schizoaffective disorder and schizophrenia. Genes Brain Behav.

[B17] Shi J, Wittke-Thompson JK, Badner JA, Hattori E, Potash JB, Willour VL, McMahon FJ, Gershon ES, Liu C (2008). Clock genes may influence bipolar disorder susceptibility and dysfunctional circadian rhythm. Am J Med Genet B Neuropsychiatr Genet.

[B18] Artioli P, Lorenzi C, Pirovano A, Serretti A, Benedetti F, Catalano M, Smeraldi E (2007). How do genes exert their role? Period 3 gene variants and possible influences on mood disorder phenotypes. Eur Neuropsychopharmacol.

[B19] Kripke DF, Rex KM, Ancoli-Israel S, Nievergelt CM, Klimecki W, Kelsoe JR (2008). Delayed sleep phase cases and controls. J Circadian Rhythms.

[B20] Sklar P, Smoller JW, Fan J, Ferreira MA, Perlis RH, Chambert K, Nimgaonkar VL, McQueen MB, Faraone SV, Kirby A (2008). Whole-genome association study of bipolar disorder. Mol Psychiatry.

[B21] Welcome Trust Case Control Consortium (2007). Genome-wide association study of 14,000 cases of seven common diseases and 3,000 shared controls. Nature.

[B22] Baum AE, Akula N, Cabanero M, Cardona I, Corona W, Klemens B, Schulze TG, Cichon S, Rietschel M, Nothen MM (2007). A genome-wide association study implicates diacylglycerol kinase eta (DGKH) and several other genes in the etiology of bipolar disorder. Mol Psychiatry.

[B23] Kelsoe JR, Spence MA, Loetscher E, Foguet M, Sadovnick AD, Remick RA, Flodman P, Khristich J, Mroczkowski-Parker Z, Brown JL (2001). A genome survey indicates a possible susceptibility locus for bipolar disorder on chromosome 22. Proc Natl Acad Sci USA.

[B24] Dick DM, Foroud T, Flury L, Bowman ES, Miller MJ, Rau NL, Moe PR, Samavedy N, El-Mallakh R, Manji H (2003). Genomewide linkage analyses of bipolar disorder: a new sample of 250 pedigrees from the National Institute of Mental Health Genetics Initiative. Am J Hum Genet.

[B25] Ebisawa T, Uchiyama M, Kajimura N, Mishima K, Kamei Y, Katoh M, Watanabe T, Sekimoto M, Shibui K, Kim K (2001). Association of structural polymorphisms in the human *period3 *gene with delayed sleep phase syndrome. EMBO reports.

[B26] Archer S, Robilliard DL, Skene DJ, Smits M, Williams A, Arendt J, von Schantz M (2003). A length polymorphism in the circadian clock gene *per3 *is linked to delayed sleep phase syndrome and extreme diurnal preference. Sleep.

[B27] Purcell S, Neale B, Todd-Brown K, Thomas L, Ferreira MA, Bender D, Maller J, Sklar P, de Bakker PI, Daly MJ (2007). PLINK: A Tool Set for Whole-Genome Association and Population-Based Linkage Analyses. Am J Hum Genet.

[B28] Brown FM (1993). Psychometric equivalence of an improved Basic Language Morningness (BALM) Scale using industrial population within comparisons. Ergonomics.

[B29] Vink JM, Groot AS, Kerkhof GA, Boomsma DI (2001). Genetic analysis of morningness and eveningness. Chronobiol Int.

[B30] Koskenvuo M, Hublin C, Partinen M, Heikkila K, Kaprio J (2007). Heritability of diurnal type: a nationwide study of 8753 adult twin pairs. J Sleep Res.

[B31] Hur YM, Bouchard TJ, Lykken DT (1998). Genetic and environmental influence on morningness-eveningness. Pers Individ Dif.

[B32] Levinson DF, Zubenko GS, Crowe RR, DePaulo RJ, Scheftner WS, Weissman MM, Holmans P, Zubenko WN, Boutelle S, Murphy-Eberenz K (2003). Genetics of recurrent early-onset depression (GenRED): design and preliminary clinical characteristics of a repository sample for genetic linkage studies. Am J Med Genet B Neuropsychiatr Genet.

[B33] Rush AJ, Trivedi MH, Ibrahim HM, Carmody TJ, Arnow B, Klein DN, Markowitz JC, Ninan PT, Kornstein S, Manber R (2003). The 16-item Quick Inventory of Depressive Symptomatology (QIDS), clinical rating (QIDS-C), and self-report (QIDS-SR): a psychometric evaluation in patients with chronic major depression. Biol Psychiatry.

[B34] Beutler E, Gelbart T (2000). Large-scale screening for HFE mutations: methodology and cost. Genet Test.

[B35] Storey JD, Tibshirani R (2003). Statistical significance for genomewide studies. Proc Natl Acad Sci USA.

[B36] Katzenberg D, Young T, Finn L, Lin L, King DP, Takahashi JS, Mignot E (1998). A clock polymorphism associated with human diurnal preference. Sleep.

[B37] Benedetti F, Radaelli D, Bernasconi A, Dallaspezia S, Falini A, Scotti G, Lorenzi C, Colombo C, Smeraldi E (2008). Clock genes beyond the clock: CLOCK genotype biases neural correlates of moral valence decision in depressed patients. Genes Brain Behav.

[B38] Kumar RA, McGhee KA, Leach S, Bonaguro R, Maclean A, Aguirre-Hernandez R, Abrahams BS, Coccaro EF, Hodgins S, Turecki G (2008). Initial association of NR2E1 with bipolar disorder and identification of candidate mutations in bipolar disorder, schizophrenia, and aggression through resequencing. Am J Med Genet B Neuropsychiatr Genet.

[B39] Liu AC, Tran HG, Zhang EE, Priest AA, Welsh DK, Kay SA (2008). Redundant function of REV-ERBalpha and beta and non-essential role for Bmal1 cycling in transcriptional regulation of intracellular circadian rhythms. PLoS Genet.

[B40] Roybal K, Theobold D, Graham A, Dinieri JA, Russo SJ, Krishnan V, Chakravarty S, Peevey J, Oehrlein N, Birnbaum S (2007). Mania-like behavior induced by disruption of CLOCK. Proc Natl Acad Sci USA.

[B41] McDearmon EL, Patel KN, Ko CH, Walisser JA, Schook AC, Chong JL, Wilsbacher LD, Song EJ, Hong HK, Bradfield CA (2006). Dissecting the functions of the mammalian clock protein BMAL1 by tissue-specific rescue in mice. Science.

[B42] Hampp G, Ripperger JA, Houben T, Schmutz I, Blex C, Perreau-Lenz S, Brunk I, Spanagel R, hnert-Hilger G, Meijer JH (2008). Regulation of monoamine oxidase a by circadian-clock components implies clock influence on mood. Curr Biol.

[B43] Greenwood TA, Schork NJ, Eskin E, Kelsoe JR (2006). Identification of additional variants within the human dopamine transporter gene provides further evidence for an association with bipolar disorder in two independent samples. Mol Psychiatry.

[B44] Berk M, Dodd S, Kauer-Sant'anna M, Malhi GS, Bourin M, Kapczinski F, Norman T (2007). Dopamine dysregulation syndrome: implications for a dopamine hypothesis of bipolar disorder. Acta Psychiatr Scand Suppl.

[B45] Yin L, Wang J, Klein PS, Lazar MA (2006). Nuclear receptor Rev-erbα is a critical lithium-sensitive component of the circadian clock. Science.

[B46] Gyulai L, Bauer M, Bauer MS, Garcia-Espana F, Cnaan A, Whybrow PC (2003). Thyroid hypofunction in patients with rapid-cycling bipolar disorder after lithium challenge. Biol Psychiatry.

[B47] Nierenberg AA, Fava M, Trivedi MH, Wisniewski SR, Thase ME, McGrath PJ, Alpert JE, Warden D, Luther JF, Niederehe G (2006). A comparison of lithium and T(3) augmentation following two failed medication treatments for depression: a STAR*D report. Am J Psychiatry.

[B48] Liu C, Li S, Liu T, Borjigin J, Lin JD (2007). Transcriptional coactivator PGC-1alpha integrates the mammalian clock and energy metabolism. Nature.

[B49] Kripke DF, Mullaney DJ, Atkinson M, Wolf S (1978). Circadian rhythm disorders in manic-depressives. Biol Psychiatry.

[B50] Albrecht U, Zheng B, Larkin D, Sun ZS, Lee CC (2001). *mPer1 *and *mPer2 *are essential for normal resetting of the circadian clock. J Biol Rhythms.

[B51] Stranger BE, Forrest MS, Dunning M, Ingle CE, Beazley C, Thorne N, Redon R, Bird CP, de GA, Lee C (2007). Relative impact of nucleotide and copy number variation on gene expression phenotypes. Science.

[B52] Knippschild U, Gocht A, Wolff S, Huber N, Lohler J, Stoter M (2005). The casein kinase 1 family: participation in multiple cellular processes in eukaryotes. Cell Signal.

[B53] Gallego M, Virshup DM (2007). Post-translational modifications regulate the ticking of the circadian clock. Nat Rev Mol Cell Biol.

[B54] Dick DM, Foroud T, Flury L, Bowman ES, Miller MJ, Rau NL, Moe PR, Samavedy N, El-Mallakh R, Manji H (2003). Genomewide linkage analyses of bipolar disorder: a new sample of 250 pedigrees from the National Institute of Mental Health Genetics Initiative. Am J Hum Genet.

[B55] Johansson C, Jansson M, Linner L, Yuan Q-P, Pedersen NL, Blackwood D, Barden N, Kelsoe J, Schalling M (2001). Genetics of affective disorders. European Neuropsychopharmacology.

[B56] Partonen T, Treutlein J, Alpman A, Frank J, Johansson C, Depner M, Aron L, Rietschel M, Wellek S, Soronen P (2007). Three circadian clock genes Per2, Arntl, and Npas2 contribute to winter depression. Ann Med.

